# Protective Effects of Circulating TIMP3 on Coronary Artery Disease and Myocardial Infarction: A Mendelian Randomization Study

**DOI:** 10.3390/jcdd9080277

**Published:** 2022-08-18

**Authors:** Heng Chen, Siyuan Chen, Hengni Ye, Xiaogang Guo

**Affiliations:** 1 Department of Cardiology, The First Affiliated Hospital, Zhejiang University School of Medicine, 79 Qingchun Road, Hangzhou 310003, China; 2 Stomatology Hospital, School of Stomatology, Zhejiang University School of Medicine, Zhejiang Provincial Clinical Research Center for Oral Diseases, Key Laboratory of Oral Biomedical Research of Zhejiang Province, Cancer Center of Zhejiang University, Hangzhou 310003, China

**Keywords:** Mendelian randomization, causal association, TIMP3, coronary artery disease, myocardial infarction

## Abstract

Tissue inhibitor of metalloproteinase 3 (TIMP3) is a protease with high expression levels in the heart and plays an essential role in extracellular matrix turnover by maintaining equilibrium with matrix metalloproteinases. Considerable data in experimental models have demonstrated a protective role of TIMP3 in coronary artery disease (CAD) and myocardial infarction (MI). However, causality remains unexplored in population studies. Here, we sought to decipher the potential causality between TIMP3 and CAD/MI using the Mendelian randomization (MR) method. We extracted summary−level datasets for TIMP3 and CAD/MI from the genome−wide association studies performed in the KORA study and CARDIoGRAMplusC4D consortium, respectively. Seven independent SNPs were obtained as instrumental variables for TIMP3. The MR analyses were replicated using FinnGen datasets, and the main results were combined in meta−analyses. Elevated genetically predicted serum TIMP3 levels were causally associated with a lower risk of CAD [odds ratio (OR), 0.97; 95% confidence interval (CI), 0.95, 0.98; *p* = 5.29 × 10^−5^] and MI (OR, 0.96; 95% CI, 0.95, 0.98; *p* = 3.85 × 10^−5^). The association patterns persisted in the meta−analyses combining the different datasets (CAD: OR, 0.97; 95% CI, 0.96, 0.99; *p* = 4.37 × 10^−5^; MI: OR, 0.97; 95% CI, 0.96, 0.99; *p* = 9.96 × 10^−5^) and was broadly consistent across a set of complementary analyses. Evidence of heterogeneity and horizontal pleiotropy was limited for all associations considered. In conclusion, this MR study supports inverse causal associations between serum TIMP3 and the risk of CAD and MI. Strategies for raising TIMP3 levels may offer new avenues for the prevention strategies of atherosclerotic cardiovascular diseases.

## 1. Introduction

Coronary artery disease (CAD) refers to a complex heart condition that often leads to ischemic heart disease and myocardial infarction (MI). Globally, deaths caused by CAD and MI have increased significantly in the past few decades, despite a set of risk factors having been identified such as smoking, hypertension, and dyslipidemia [1,2]. Recognition of the protective factors for CAD and MI may offer new avenues for better preventive and treatment strategies.

Tissue inhibitors of metalloproteinases (TIMPs), consisting of TIMP1, TIMP2, TIMP3, and TIMP4, are endogenous protein regulators of matrix metalloproteinases (MMPs) [3]. The MMPs/TIMPs balance plays a critical role in extracellular matrix (ECM) turnover and tissue fibrosis. Among the four TIMPs, TIMP3 is unique due to its broader inhibitory spectrum against metalloproteinases and tight binding to ECM [4,5,6]. More importantly, its involvement in cardiovascular diseases has been widely reported. TIMP3 was highly expressed in heart tissue [7] but was remarkably downregulated in the animal MI models as well as in human subjects with ischemic cardiomyopathy [8,9]. TIMP3−deficient mice exhibited compromised cardiac function, maladaptive cardiac remodeling, and higher mortality after MI by coronary ligation than wild−type controls [10,11]. Moreover, the targeted overexpression of TIMP3 by adenovirus or injectable hydrogels attenuated infarct expansion and improved cardiac function after MI in animal models [8,12]. However, it remains unestablished whether these observations can be translated into prevention or treatment strategies in clinical settings; few clinical studies have investigated the potential effect of TIMP3 on CAD or MI. Data from large−scale randomized controlled trials (RCT) may shed light on this issue but at a relatively high cost.

As one of the epidemiological methods, Mendelian randomization (MR) has flourished in recent years. Its core idea is to use genetic variants that follow the Mendelian law of heredity as proxies for the traits of interest [13,14]. Therefore, MR creates a similar scenario to RCT where residual confounders and reverse causality are largely avoided and causal association is determined [13,14]. A two−sample MR design relies on the genetic datasets of two independent populations for exposure and outcome. To further our knowledge of the potential protective effect of TIMP3 on atherosclerotic cardiovascular diseases (CVDs) in the European population, we applied a two−sample MR approach with replication of the main analysis using different datasets and a set of complementary methods.

## 2. Materials and Methods

### 2.1. Study Design

**Figure 1:**
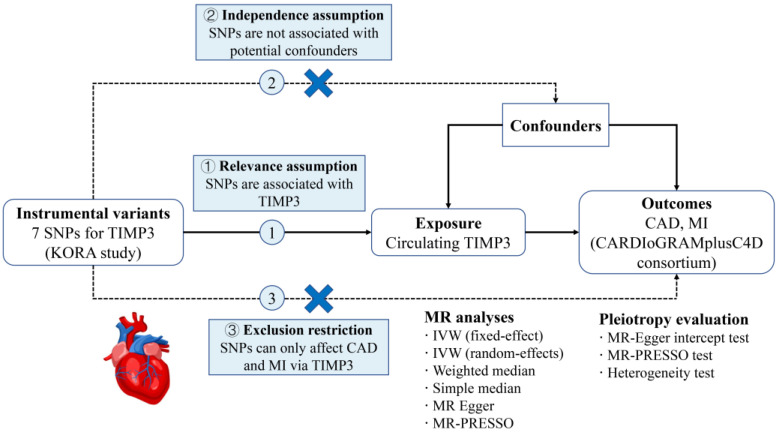
Schematic diagram of the present Mendelian randomization study. SNPs, single−nucleotide polymorphisms; TIMP3, tissue inhibitors of matrix metalloproteinase 3; CAD, coronary artery disease; MI, myocardial infarction; KORA, Cooperative Health Research in the Region of Augsburg; CARDIoGRAMplusC4D, Coronary Artery Disease Genome−Wide Replication and Meta−Analysis plus the Coronary Artery Disease Genetics; IVW, inverse−variance weighted; MR−PRESSO, MR Pleiotropy Residual Sum and Outlier.

Single−nucleotide polymorphisms (SNPs) were selected as the instrumental variables (IVs) for circulating TIMP3 from a genome−wide association study (GWAS) dataset [15]. Their associations with CAD and MI were made available in another GWAS meta−analysis [16]. Three key assumptions of the two−sample MR design were fulfilled in this study (Figure 1) [17]. This study followed the Strengthening the Reporting of Observational Studies in Epidemiology Using Mendelian Randomization (STROBE−MR) statement (Table S1) [18].

All the datasets used in this study are publicly available. Ethical permits and written informed consent were provided in their original studies.

### 2.2. Data Sources and IV Selection

We selected SNPs associated with TIMP3 from the Cooperative Health Research in the Region of Augsburg (KORA) study that investigated blood samples from 997 individuals of European ancestry (adjusted for age, gender, and body mass index) [15]. The associations of these SNPs with CAD and MI were available in a large GWAS meta−analysis conducted by the Coronary Artery Disease Genome−Wide Replication and Meta−Analysis plus the Coronary Artery Disease Genetics (CARDIoGRAMplusC4D) consortium. The meta−analysis comprised 48 RCTs and population−based studies and included 184,305 individuals (CAD: 60,801 cases; MI: 43,676 cases), predominantly (77%) of European descent (Table 1) [16]. CAD was diagnosed based on a broad standard that included MI, acute coronary syndrome, chronic stable angina, and coronary stenosis >50% [16]. Additionally, we replicated the analyses using the outcome datasets from the FinnGen consortium, which consisted of 25,707 CAD cases and 15,787 MI cases (Table 1) [19]. Here, CAD was defined by the International Classification of Diseases (ICD)−8, code 410 or 4110; ICD−9, code 410 or 4110; and ICD−10, code I20.0, I21, or I22. MI was diagnosed by ICD−8, code 410; ICD−9, code 410; and ICD−10, code I21 or I22.

In total, 41 SNPs associated with TIMP3 at a *p* level of <1 × 10^−5^ were provided in the KORA study [15]. Here, valid IVs were selected under the following quality−control steps. First, we set a genome−wide significance at *p* < 5 × 10^−8^, leaving 33 SNPs that satisfied the first key assumption (relevance assumption). Then, we performed a clumping process (r^2^, 0.1; clumping window, 10,000 kb) [20] with reference to the 1000 genomes European panel [21] to avoid the influence of linkage disequilibrium. For the third key assumption (exclusion restriction assumption), we performed the MR Steiger filtering test to identify the SNPs suggestive of causality in the reverse direction [22] and remove these SNPs, if any (Table S2). In addition, the phenotypes associated with these SNPs were searched across PhenoScanner V2 [23]. Here, we found that none of these IVs were significantly associated (*p* < 5 × 10^−8^) with potential confounders (i.e., risk factors for CAD/MI). Finally, as shown in Table S3, 7 SNPs were chosen as the IVs for the subsequent MR analyses. All these SNPs carried a minor allele frequency (MAF) higher than 0.01, suggesting minor statistical bias from low confidence.

### 2.3. Statistical Analysis

As displayed in Figure 1, the inverse−variance weighting (IVW) approach in the fixed−effect model was utilized as the primary statistical model where SNP−specific Wald−ratio estimates were pooled [24]. This conventional MR approach provides convincing results when all the IVs are valid and do not exhibit pleiotropic effects (from IVs to CAD/MI, bypassing TIMP3). Consequently, a set of complementary analyses were applied in scenarios with different states of pleiotropy, including the IVW in the random−effects model [25], weighted median [26], MR−Egger [27], and MR Pleiotropy Residual Sum and Outlier (MR−PRESSO) [28] methods. The IVW in the random−effects model can yield more reliable estimates in the presence of high heterogeneity among SNPs [29]. The weighted median method assumes that more than 50% of the weights are derived from valid IVs [26]. The MR−Egger analysis can identify unbalanced, horizontal pleiotropy through its intercept test [30]. Also, this approach allows causal estimates to be generated after correction for pleiotropic effects, albeit with impaired statistical power [27]. We additionally used the MR−PRESSO method to identify pleiotropic outliers. This method returns relatively unbiased estimates after excluding outlier SNPs if present [28]. Heterogeneity among IVs was tested by calculating Cochran’s Q and I^2^ statistics in the IVW model. In addition to the MR−Egger analysis, horizontal pleiotropy was further explored using the MR−PRESSO global test [28] and Phenoscanner V2 (as mentioned above). Scatter plots describing the causal effects of genetically predicted TIMP3 on CAD and MI were also provided.

MR estimates across CARDIoGRAMplusC4D and FinnGen were combined using meta−analyses in the fixed−effect model. Phenotypic variance in TIMP3 explained by each IV (R^2^) was calculated using a commonly used formula: R^2^ = 2 × EAF × (1 − EAF) × beta^2/(2 × EAF × (1 − EAF) × beta^2) + 2 × EAF × (1 − EAF) × se × N × beta^2). Here, EAF, beta, se, and N represent the effect allele frequency, effect size, standard error, and sample size, respectively [31,32]. Power calculations were performed with the online tool mRnd based on the outcome sample size, proportion of cases, R^2^ sum, and a type I error rate of 0.05 (Table S4) [33]. Given the multiple analyses, associations with a Bonferroni−corrected *p*−value of <0.0125 were considered significant. The odds ratios (ORs) of the associations were scaled to per log increase in genetically determined TIMP3 levels. All the MR analyses in this study were conducted in software R (version 4.1.0) [34] with R packages including TwoSampleMR [35] and MR−PRESSO [28].

## 3. Results

The results for the MR Steiger filtering test are demonstrated in Table S2. All 7 TIMP3−associated genetic variants explained more variance in TIMP3 than in CAD or MI, suggesting causality in the expected direction (Table S2). These SNPs collectively explained 12.8% of the phenotype variance of TIMP3 (Table S3). No sample overlap was found between the data sources for TIMP3 and CAD/MI (Table 1).

**Figure 2:**
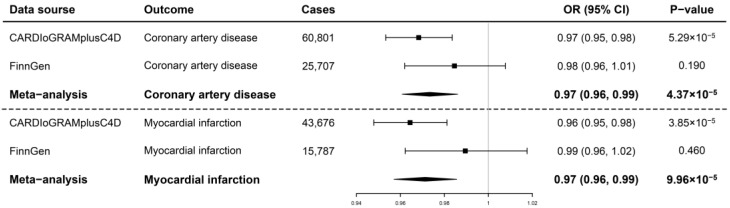
Association of genetically predicted serum TIMP3 levels with risk of CAD and MI. TIMP3, tissue inhibitors of matrix metalloproteinase 3; CARDIoGRAMplusC4D, Coronary Artery Disease Genome−Wide Replication and Meta−Analysis plus the Coronary Artery Disease Genetics; OR, odds ratio; CI, confidence interval.

**Figure 3:**
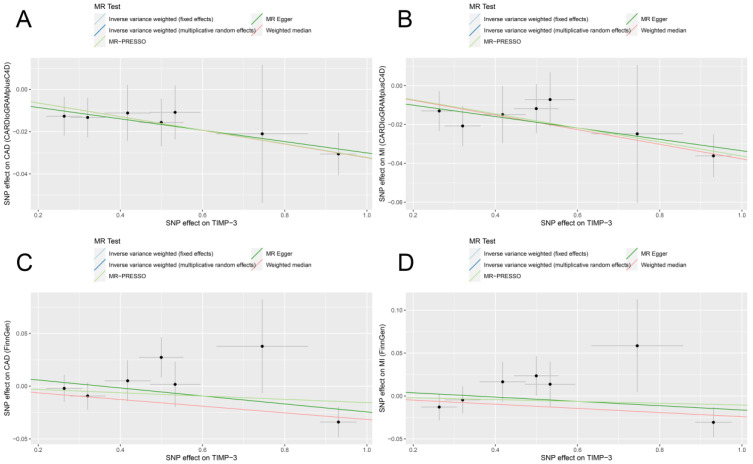
Scatter plot of the MR estimates for the association of serum TIMP3 levels with risk of CAD and MI based on CARDIoGRAMplusC4D (**A**,**B**) and FinnGen (**C**,**D**). TIMP3, tissue inhibitors of matrix metalloproteinase 3; CAD, coronary artery disease; MI, myocardial infarction; CARDIoGRAMplusC4D, Coronary Artery Disease Genome−Wide Replication and Meta−Analysis plus the Coronary Artery Disease Genetics; MR−PRESSO, MR Pleiotropy Residual Sum and Outlier. Dots refer to SNPs.

In the fixed−effect IVW model, the per log increase in the circulating TIMP3 levels conferred a protective effect on CAD (OR, 0.97; 95% confidence interval (CI), 0.95, 0.98; *p* = 5.29 × 10^−5^) and MI (OR, 0.96; 95% CI, 0.95, 0.98; *p* = 3.85 × 10^−5^) based on the CARDIoGRAMplusC4D datasets (Figure 2 and Figure 3). The estimates based on the FinnGen datasets were each positive, albeit with a reduced magnitude and noticeably wider CIs (Figure 2 and Figure 3). The protective effects were supported by a meta−analysis combining the CARDIoGRAMplusC4D and FinnGen datasets (CAD: OR, 0.97; 95% CI, 0.96, 0.99; *p* = 4.37 × 10^−5^; MI: OR, 0.97; 95% CI, 0.96, 0.99; *p* = 9.96 × 10^−5^; Figure 2 and Figure 3). The robustness of the main results was reinforced by a series of complementary analyses of the different assumptions, where causality was oriented in the same direction as the main analysis (Table 2). Cautions were warranted here since several associations did not reach the Bonferroni−corrected *p*−value of 0.0125 (Table 2).

Cochran’s Q and I^2^ statistics demonstrated a low heterogeneity degree among the SNPs for the TIMP3–CAD and TIMP3–MI associations (P_Cochran’s Q_ > 0.0125 and I^2^ < 25%; Table 3). No substantial horizontal pleiotropy was detected for both outcomes, as suggested by the MR−Egger intercept *p* and MR−PRESSO global test *p* (P_intercept_ > 0.0125; P_global test_ > 0.0125; Table 3). In addition, we found no pleiotropic SNP to be corrected with the MR−PRESSO outlier test. This study had over 80% power to detect an OR of 1.039 (or 0.961) for CAD and 1.043 (or 0.957) for MI based on the CARDIoGRAMplusC4D consortium, and an OR of 1.052 (or 0.948) for CAD and 1.065 (or 0.935) for MI based on the FinnGen consortium (Table S4).

## 4. Discussion

In this study, we used the two−sample MR framework by applying public data from different GWAS to decipher the causality between TIMP3 and the risk of CAD and MI. We provided genetic evidence showing that increased circulating TIMP3 levels lead to a lower risk of CAD and MI. The positive results remained broadly concordant across a set of complementary analyses. No heterogeneity or horizontal pleiotropy was detected. Although replication studies using clinical study designs are necessary, our findings suggested that targeting higher levels of TIMP3 may have implications for better primary preventive strategies for these conditions. 

MMPs have been widely recognized as a regulator for the balance of ECM degeneration and synthesis during wound healing post−MI [36]. It is not only a biomarker but also a key driver in the development of MI and post−MI remodeling [36]. This has prompted investigators to explore whether the inhibitors of MMPs (MMPi) can prevent atherosclerosis and MI and mitigate MI injury. For example, it was reported that RXP470.1 (a selective MMP−12i) slowed atherosclerotic plaque development [37] in mice; rabbits randomized to CP−471,474 (a broad−spectrum MMPi) exhibited attenuated ventricular remodeling post−MI [38]; and TIMP3, an endogenous and broad−spectrum MMPi, allowed smaller atherosclerotic plaques [39] and reduced infarct expansion after MI [8] in animal models. However, clinical trials regarding the potential effects of MMPi on CAD or MI were limited. Only one oral MMP inhibitor (PG−116800) was tested with RCT (PREMIER, Prevention of Myocardial Infarction Early Remodeling), where 253 subjects with MI and heart failure were enrolled and were randomly assigned PG−116800 or a placebo for three months [40]. The trial revealed that PG−116800 could not attenuate left ventricular dysfunction or clinical outcomes [40]. This could be attributed to the insufficient dose or the fact that this treatment strategy is inherently ineffective in humans after the onset of MI, despite being effective in animal studies. On the other hand, it would be interesting to figure out whether MMPi exerts a protective effect contributing to a lower risk of CAD and MI through population studies. This MR analysis sought to illustrate this issue by utilizing datasets from GWASs of the European population. The MR design and enlarged sample size of the GWASs strengthened the causal inference. Here, our approach revealed a causal association between TIMP3 and the risk of CAD and MI. Given that atherosclerosis is the pathophysiological mechanism of CAD and MI, our results corroborate the findings from the basic research showing a protective effect of TIMP3 on atherosclerosis [39,41]. These findings enhance the basis for further studies to evaluate the clinical relevance of rising TIMP3 levels for preventing CAD and MI.

The increased inflammatory level is one of the well−established players in the onset of atherosclerosis and the development of atherosclerotic CVDs. It was reported that TIMP3 knockout led to the accumulation of macrophages in the aortic root, pro−inflammatory macrophage polarization, and increased serum levels of inflammatory marker (MCP1) [42]. Mice with TIMP3 deficiency exhibited increased TNF−a activity in the myocardium before and after MI by coronary ligation [11]. In addition to targeting inflammation, TIMP3 exerts its protective effect against atherosclerosis by regulating endothelial function and extracellular matrix remodeling. Inhibition of multiple proteases, such as MMP 2/9 and disintegrin metalloproteinase (ADAM) 10/17, have been implicated in the protection afforded by TIMP3 [43,44]. Treatment with broad−spectrum MMPi can primarily ameliorate the cardiac injuries observed in animal models with TIMP3 deficiency [10,45].

The MR framework is one of the significant strengths of this study, which reduces common biases in observational studies such as residual confounders and reverse causality. Secondly, no heterogeneity or horizontal pleiotropy was found for all outcomes considered using sensitive analyses including MR−Egger regression as well as the MR−PRESSO global test. The MR Steiger test and Phenosacanner V2 were used with no pleiotropic SNPs detected as well. Therefore, the risk of pleiotropic effects should be minimal in this study. The meta−analyses that combined the MR estimates based on different datasets further strengthened the causal inference.

However, limitations still exist and need consideration. First, the potential nonlinear associations between TIMP3 and CAD/MI were not assessed since this study was based on summary−level data. Second, the statistical power for the main analyses did not reach a threshold of 80%. This could be due to the slight variance of TIMP3 explained by the selected IVs. Therefore, cautions are warranted for the interpretation of the results. Third, a low degree of heterogeneity existed in the meta−analyses of the main results, which could be attributed to the different definitions of CAD and MI between the CARDIoGRAMplusC4D and FinnGen consortium. Moreover, population stratification may introduce bias since the CardiogramplusC4D consortium included individuals of non−European (approximately 23%) descent. In addition, there is limited generalizability of the results to other populations. Finally, accumulating evidence from basic research suggested that TIMP3 exerted a protective effect against cardiac injury post−MI [8,12,46]. However, this study cannot evaluate the prognostic impact of TIMP3 on MI due to the MR design. Future longitudinal research may shed light on this issue.

## 5. Conclusions

This MR study found genetic evidence showing that higher serum TIMP3 levels can lead to a lower tendency to suffer from CAD and MI. These findings suggest that prevention strategies to replenish TIMP3 may provide a protective outcome in atherosclerotic CVDs.

## Figures and Tables

**Table 1:** Detailed information of studies and datasets used for analyses.

Data Source	Phenotype	Sample Size	Cases	Population	Adjustment
KORA study	TIMP3	997	/	European	Age, gender, and body mass index
CARDIoGRAMplusC4D	CAD	184,305	60,801	77% European	Not reported
	MI	184,305	43,676		
FinnGen	CAD	260,405	25,707	European	Age, sex, and up to 20 genetic principal components
	MI	238,338	15,787		

KORA, Cooperative Health Research in the Region of Augsburg; CARDIoGRAMplusC4D, Coronary Artery Disease Genome−Wide Replication and Meta−Analysis plus the Coronary Artery Disease Genetics; CAD, coronary artery disease; MI, myocardial infarction.

**Table 2:** Association of genetically predicted circulating TIMP3 levels with CAD and MI risk in complementary analyses.

Data Source	Outcome	SNPs, *n*	Method	OR	95% CI	*p*−Value
CARDIoGRAMplusC4D	CAD	7	IVW (random−effects)	0.97	0.96, 0.97	7.26 × 10^−38^
		7	Weighted median	0.97	0.95, 0.99	8.30 × 10^−3^
		7	MR−Egger	0.97	0.94, 1.01	0.204
		7	MR−PRESSO *	0.97	0.96, 0.97	1.36 × 10^−5^
CARDIoGRAMplusC4D	MI	7	IVW (random−effects)	0.96	0.95, 0.97	4.49 × 10^−13^
		7	Weighted median	0.96	0.94, 0.99	7.58 × 10^−3^
		7	MR−Egger	0.97	0.93, 1.01	0.208
		7	MR−PRESSO *	0.96	0.95, 0.97	3.53 × 10^−4^
FinnGen	CAD	7	IVW (random−effects)	0.98	0.96, 1.01	0.226
		7	Weighted median	0.97	0.94, 1.00	0.027
		7	MR−Egger	0.96	0.91, 1.02	0.252
		7	MR−PRESSO *	0.98	0.96, 1.01	0.272
FinnGen	MI	7	IVW (random−effects)	0.99	0.96, 1.02	0.469
		7	Weighted median	0.98	0.94, 1.01	0.166
		7	MR−Egger	0.98	0.91, 1.04	0.503
		7	MR−PRESSO *	0.99	0.96, 1.02	0.497

CARDIoGRAMplusC4D, Coronary Artery Disease Genome−Wide Replication and Meta−analysis plus the Coronary Artery Disease Genetics; CAD, coronary artery disease; MI, myocardial infarction; SNPs, single−nucleotide polymorphisms; IVW, inverse−variance weighted; MR−Egger, Mendelian randomization−Egger; MR−PRESSO, MR−pleiotropy residual sum and outlier; OR, odds ratio; CI, confidence interval. * No outliers detected.

**Table 3:** Evaluation of heterogeneity and directional pleiotropy using different methods.

Data Source	Outcome	Heterogeneity		Pleiotropy	
		I^2^ (%)	Cochran’s Q *p*	MR−Egger Intercept *p*	MR−PRESSO Global Test *p*
CARDIoGRAMplusC4D	CAD	0	0.925	0.722	0.997
	MI	0	0.997	0.761	0.946
FinnGen	CAD	18	0.315	0.432	0.319
	MI	4	0.395	0.660	0.367

CARDIoGRAMplusC4D, Coronary Artery Disease Genome−Wide Replication and Meta−analysis plus the Coronary Artery Disease Genetics; CAD, coronary artery disease; MI, myocardial infarction; MR−Egger, Mendelian randomization−Egger; MR−PRESSO, MR−pleiotropy residual sum and outlier.

## Data Availability

All the datasets used in the present study are publicly available. The data generated or analyzed during this study have been included in this published article [and its Supplementary Information files].

## Supplementary Materials

The following supporting information can be downloaded at: https://www.mdpi.com/article/10.3390/jcdd9080277/s1. Table S1: STROBE−MR Checklist. Table S2: Results of MR Steiger direction test. Table S3: Genome−wide significant SNPs associated with TIMP3 and their association with CAD and MI. Table S4: Power calculations in Mendelian randomization study of TIMP3 and risk of CAD and MI.
